# Mishaps, errors, and cognitive experiences: on the conceptualization of perceptual illusions

**DOI:** 10.3389/fnhum.2015.00190

**Published:** 2015-04-13

**Authors:** Daniele Zavagno, Olga Daneyko, Rossana Actis-Grosso

**Affiliations:** ^1^Department of Psychology, University of Milano-BicoccaMilano, Italy; ^2^Department of Neuroscience, University of ParmaParma, Italy

**Keywords:** perceptual illusions, gestalt theory, cognitivism, ecological approach, veridicality, reality, perceptual theories

## Abstract

Although a visual illusion is often viewed as an amusing trick, for the vision scientist it is a question that demands an answer, which leads to even more questioning. All researchers hold their own chain of questions, the links of which depend on the very theory they adhere to. Perceptual theories are devoted to answering questions concerning sensation and perception, but in doing so they shape concepts such as reality and representation, which necessarily affect the concept of illusion. Here we consider the macroscopic aspects of such concepts in vision sciences from three classic viewpoints—Ecological, Cognitive, Gestalt approaches—as we see this a starting point to understand in which terms illusions can become a tool in the hand of the neuroscientist. In fact, illusions can be effective tools in studying the brain in reference to perception and also to cognition in a much broader sense. A theoretical debate is, however, mandatory, in particular with regards to concepts such as *veridicality* and *representation*. Whether a perceptual outcome is considered as veridical or illusory (and, consequently, whether a class of phenomena should be classified as perceptual illusions or not) depends on the meaning of such concepts.

## Introduction

To survive in an ever-changing environment, animals must gather information about their surroundings, for animals must seek nutrition, mate, find shelter, etc. To carry out its tasks an animal must rely on information collected through its sensory systems. This must be “extracted” and “abstracted” (Gibson, 1979) from an abundance of sensory input, and ultimately understood. Whatever one’s theoretical stance, the aforementioned operations can be considered in terms of processes—registration of structural invariants (Gibson, 1979), information elaboration driven by assumptions (Rock, 1983), functional organization of stimuli (Koffka, 1935), etc.—which ultimate purpose is to generate a dynamic *model* of the surrounding environment, on the basis of which the animal acts.

Though our sketch is quick and rudimental it should still satisfy most scientists interested in the workings of perceptual and cognitive systems. Yet, as soon as one starts to consider *how* sensory data is processed and the *likes* of the model generated, things become convoluted. We here discuss how visual models of the environment are conceptualized in psychology, which will allow us to put into focus how illusions are conceived by most vision scientists, and briefly discuss the role they may play in current vision sciences. As *facts*, illusions are theoretically neutral, yet the way they are described and handled in perceptual studies is largely dependent on the theoretical frameworks of scientists. Even a very simple classification system of visual illusions encompasses to some minimum extent a level of explanation (Vicario, 2011). We will not deal with such systems here, as we are interested in visual illusions as a general concept.

## Theoretical Frameworks

The term “model” is itself troublesome, as it can denote two things: a *replica* or a *representation* of macro aspects of the environment. These two meanings, as we intend them here, are mutually exclusive. A replica means that the model is in all ways a faithful rendering of the macro aspects of something else. A model conceived in terms of a representation is instead a mediated set of assumptions or a functional scheme about aspects of the environment (events, objects, environmental layout) that are potentially relevant to the perceiving organism. The difference between those conceptualizations is better understood if we consider the term *veridicality*. If something is classified as veridical, it means that it is truthful, adherent to “reality”, thus not *illusory*.

For a model conceived as a *replica*, the term “veridicality” has no special meaning: it adds nothing to the concept as the model is already conceived in terms of an exact duplicate of what it models. A replica is all, or else it is not a replica. This is, in sum, the stance of the Ecological theory of visual perception (Gibson, 1979). To the famous question posed by Koffka (1935, p. 75) *Why do things look as they do*, a Gibsonian would likely answer *Because they are what they are*. According to this theoretical approach, the visual environment replicates the macro aspects of the physical environment: we see what is there, not more, not less. For instance, elements of the environment directly offer information in terms of their possible uses. There is no need to generate hypotheses to *see* that we can use a concave object to collect water from a pond, if we needed to. Gibson spoke about *affordances*—a concept he modified from Lewin’s (1926) *Aufforderungscharakter*—in terms of invariant properties of physical objects that specify how they can be employed. No inferences are required: the visual system extracts and abstracts invariants of structure from the proximal stimulus. Veridicality has no place as a term in the lexicon of a Gibsonian because *things* *appear exactly as they should*.

If the model is instead conceived in terms of a representation of the environment, then rather than a *copy* it is a *reconstruction* of the physical environment triggered by information entering the visual system. Conceiving the model as a representation, however, can lead to different directions. For instance a representation can be *realistic* or *functional*. Veridicality is fundamental in the first case, irrelevant in the second.

Conceiving the model in terms of a *veridical* *representation* implies that the goal of the visual system is to *represent* relevant aspects of the physical environment as faithful as possible. A veridical representation, however, is not a mere copy, it is an *interpretation* of the outside world based on visual information (cues) gathered by the system and combined with content already stored in the brain. The processes behind the determination of the model are generally referred to as rapid unconscious inferences (Gregory, 1995) and in recent years have been elegantly accounted for in terms of Bayesian inferences (Knill et al., 1996). This is, in sum, the stance of cognitive approaches to visual perception. The hypothesis that drives such theories is that sensory data are largely undetermined, meaning that sensory input is basically ambiguous in terms of what it may refer to. Hence, the model generated by the visual system is a representation largely dependent on unconscious inferences driven by past experience, information integration, etc. The result is a cogent visual environment tightly related to the physical environment that builds on three important factors: information *density* (quantitative and qualitative characteristics), the observer’s *experience* (past or genetically encoded) and *expectations* (contextual). For instance, the Müller-Lyer illusion can be accounted for in terms of inappropriate constancy scaling (Gregory, 1963): the pair of angles placed at the ends of each line are implicit depth cues that induce the visual system to interpret the lines as belonging to different depth planes. Adding cues to the scene, either coherent or contrasting with the aforementioned ones, will enhance or reduce the illusion.

The difference between a veridical model and a replica is that the last is directly given as copy of the outside world, whilst the first is generated by means of assumptions and logic (Rock, 1983) governed by a likelihood principle: the visual system constructs the interpretation that is the most likely state of the environment that could have caused the sensory input.

Conceiving the model in terms of a *functional representation* of the surrounding environment means that the representation is functional to the needs of the person, and whether it is veridical or an exact replica of the environment becomes an irrelevant issue. The model is guaranteed to work not because it replicates the physical environment in all its macro aspects, nor because the visual system infers through unconscious cognitive processes the actual nature and qualities of the physical environment, but because it is *egocentric*, i.e., the person is the center of the environment. This is, in sum, the stance of traditional and modern Gestalt approaches to visual perception. To work, the model must assume a somewhat deterministic set of operations on the sensory input, which the gestalt psychologists referred to in terms of auto-organization of stimuli and postulated as a gestalt identity between the perceptual experience and its underlying cortical processes (isomorphism) (Metzger, 1963; Köhler, 1971). The Gestalt and the Ecological approaches share a common indifference towards the notion of veridicality, but for different reasons: if for the Ecological approach such concept is *unnecessary*, for the Gestalt approach it is *irrelevant*, given that there is no way to establish whether a percept is veridical with respect to the distal source of stimulation, as knowledge of such source comes from the percept itself, or is mediated by the use of some instrumentation, which however cannot tell what is more veridical. Moreover physical properties of a distal stimulus are only rough correlates of what is perceived (Zavagno et al., 2011a,b). For instance, the luminance of an achromatic surface is the physical correlate of lightness (achromatic surface color), which however, does not correlate with the luminance of the physical surface: a sheet of paper with reflectance 70% may appear light gray even if its luminance changes dramatically, because lightness is a contextual experience, *functional* to the characteristics of the scene in relation to the perceiver. The same holds for other percepts: e.g., the velocity of a moving target may appear slower or faster depending on factors such as background figural features (Actis-Grosso, 2008; Actis-Grosso et al., 2008).

The differences between Gestalt and Cognitive theories are especially centered on the mechanisms involved in visual perception. It is not just by chance that Rock (1983) entitled his book *The logic of perception*, whereas Kanizsa (1980) entitled his *La grammatica del vedere* (*The grammar of seeing*): the term “logic” refers to inferential processes that share something in common with higher cognitive processes, whilst “grammar” refers to a set of structural rules that are more binding. In the first case it is the richness of information available, both in terms of actual stimulation and of stored knowledge, that drives the validity of the representation of the world. In the second case it is assumed that the representation is driven by the pattern of stimulation and the structure of the visual system.

The three groups of theories described above are “pure”: they are clearly opposed one to another. Attempts have been made to somewhat fuse them together, in particular by incorporating “what is good” from the Ecological and the Gestalt theories within Cognitive approaches. Though in principle the fusion of different perspectives enriches our understanding of phenomena and processes, in the case of visual illusions the result is often a theoretical pot-pourri that appears logical and capable of answering all questions, but in fact solves no problems.

## The Place of Illusions in Perceptual Theories

If we assume that *we see what is there* (Ecological approach), then illusions should have no place in our visual experience, because by definition illusions do not exist in the physical world. There is of course a problem: illusions are a pretty well known category of visual phenomena. Given a problem, there is also a solution: without entering details, the existence of illusions is basically resolved by considering the *richness* of information available in the environment. As the ecological validity of an environment is reduced—e.g., an experimental setup in a laboratory can be considered an environment with relatively poor ecological validity—also the richness of the information available is reduced. Illusions occur because the visual information available is relatively poor. Illusions are therefore *mishaps* to be found only in reduced settings and artificial setups, not in ecologically valid environments. From the viewpoint of the Ecological approach it is therefore almost pointless to study visual illusions, unless these become tools in studying the extraction and abstraction of invariants of structure.

If we assume that our perceptual experience is a representation of the world that tends towards veridicality (Cognitive approaches), then illusions are *errors* driven by specific sets of cues and assumptions that guide scaling processes and scene analyses. Illusions appear not only or necessarily because the information from the environment is quantitatively poor but because it is basically ambiguous. The quality of visual information is the key towards veridicality: the more non-contrasting cues are entwined, the more accurate our representation of the environment. As the number of entwined cues is reduced, and the quantity of conflicting information is eventually increased, stimulus ambiguity is also increased along with the probability of an *error* by the visual system in rendering some properties within its representation. Such errors are, however, systematic (Gilchrist, 2006) and can therefore be used to study the *logic* by which the visual system works, i.e., the set rules employed to run inferences and combine cues.

If we assume that our perceptual experience is a functional representation of the world, centered around us (Gestalt approach), then illusions do not exist from a purely perceptual stance, as we have no clue to what we should see other than what we are actually seeing. As with the Ecological approach, we seem to have a problem because illusions are a pretty well known category of phenomena. The problem is however solved by considering our actual experience of visual illusions: we are *aware* of an illusion not because we *see* it but rather because we* know* how things are, or should be, from a physical point of view. For instance, in the Müller-Lyer illusion we see two lines, one delimited by converging, the other by diverging angles, and we *see* that those two lines are different in length. If we measure the two lines, however, we then find out that they are physically equal in length; nevertheless they still appear different in length even if we *know* they are physically equal (Vicario, 2005). In other words, illusions are cognitive experiences, not purely perceptual ones: to appreciate an illusion we must have awareness of the discrepancy between our perceptual reality and the physical world; such awareness drives both on perceptual and cognitive material, but it is conflicting only at a cognitive level. This dual origin of illusions renders them useful tools in studying both perception and cognition.

## Illusions and Visual Neuroscience

Two friends are in a car; the passenger asks the driver: “Why are we going faster?” “Because I pressed on the pedal”, answers the driver. The answer is formally correct but not very informative. What does this have to do with illusions and neuroscience? The answer of *why* things appear as they do cannot be confined to the definition of the neural correlates of visual phenomena: the *where* issue is not a sufficient answer. In the past two decades a lot has been written about *where* things happen in the brain, which is an important starting point that however does not fully address the *how* and *why* issues. How processes take place will become more clear when neuroscientists will be able to connect single cell responses to networks of cells, and understand the communication and integration of information across networks. It is not only a question of mapping the brain and describing its architecture, but it is also a matter of understanding its functional architecture and interconnections. Neuroscience is already stepping on the path that leads to a fruitful understanding of how the brain processes visual information. We believe that illusions may become relevant tools in such studies, if research is not limited to finding *where* an illusion occurs in the brain, but *how* and *why* it comes to be.

The procedure required to answer *why* questions, however, cannot be disjointed from a theoretical framework, which will also condition the experimental questions to be addressed. For instance, Movshon and Blakemore (2000) claim that to understand “the full richness of sensory processing, we must appreciate both the volume of *computation* and the sophisticated *deductions* that give rise to our sensory experience” (p. 251). The approach adopted is that of a *cognitive* *neuroscience*, where the assumption is that inferences and deductions are a core feature in determining the *model* (sensory experience) generated by the visual system. This type of approach is particularly eager to find top-down neural phenomena, which in combination with bottom-up information, drives the generation of the visual model. Within this theoretical framework visual illusions can become useful tools not only for uncovering areas of the brain that are supposed to be responsible for systematic errors due to wrong assumptions, inappropriate scaling, etc, but also for studying the ongoing neural communication between different areas of the brain while perceptual processing is taking place.

Gestalt theory has also embraced the challenges of a new relationship with neuroscience, looking for Gestalt-like neural mechanisms (Ehrenstein et al., 2003; Calì, 2013). The most significant project that goes in this direction is probably *GestaltReVision* directed by Johann Wagemans, with an impressive output in terms of empirical studies connecting psychophysics to neuroscience (Mijović et al., 2014; Sassi et al., 2014). But what role can visual illusions have in a Gestalt approach to visual neurosciences? While a gestaltist would agree with Rogers (2014, p. 840) that “there is no satisfactory way of distinguishing between those aspects of our perception that we regard as veridical and those we label as illusions”, we do not think that a Gestalt approach to visual neuroscience would dismiss illusions as phenomena that “might reveal how our perceptual systems work *(or fail to work) in only rather limited or impoverished stimulus situations*” (p. 844, emphasis ours). Within a Gestalt approach, illusions become “natural laboratories” (Kanizsa, 1980) as their systematic nature can be advantageously used to uncover the functional architecture of the visual system. The goal is understanding how the visual system produces a perfectly functional model of the world that goes beyond the visual information available (Kanizsa, 1980).

What about the Ecological approach and its relationship to current visual neuroscience? Although links between the two appear to be less direct, it is a fact that current perception-action theories resonate with at least some of Gibson’s ideas, e.g., the role attributed by Gibson to vision as a support for behavior, which we believe contributed in distinguishing between vision for recognition and vision for action (Goodale and Milner, 1992; Milner and Goodale, 2008), with strong parallels between the functioning of the dorsal stream (visual control of motor behavior) and the Ecological approach to visual perception (Norman, 2002). The survival of Gibson’s legacy, however, relies especially on the concept of *affordance*, a relevant perceptual attribute in the study of perception for action and its reconciliation with a more modern approach in terms of active vision (Findlay and Gilchrist, 2003). In this respect, we believe that visual illusions, though intended as mishaps within a purely Ecological approach, may still maintain an important role in studying perception for action (Goodale and Humphrey, 1998; Bruno et al., 2008) also within vision neurosciences, as they may aid in understanding the different ways in which the brain processes spatial information based on the goals of the perceiver-actor.

Illusions have the potential to play a relevant role in visual and cognitive neuroscience; however how illusions are employed ought to reflect theoretical stances in which terms such as *veridicality* and* representation* are thoroughly defined. How the neural mechanisms that shape perception and cognition are described will inevitably impact on how *reality* is conceived. Researchers should be aware that all models rely on metaphysical assumptions, the cornerstones of which are the two faces of a same coin: reality/illusion.

## Conflict of Interest Statement

The authors declare that the research was conducted in the absence of any commercial or financial relationships that could be construed as a potential conflict of interest.
